# From microelectrode arrays to all-optical and multimodal neural interfaces: emerging platforms for spatiotemporal interrogation of *in vitro* neural circuits

**DOI:** 10.3389/fnsyn.2025.1732955

**Published:** 2025-12-09

**Authors:** Song Wang, Sarah Gordon, Chris French, Ranjith R. Unnithan, Dechuan Sun

**Affiliations:** 1 Department of Electrical and Electronic Engineering, The University of Melbourne, Melbourne, VIC, Australia; 2 The Florey Institute of Neuroscience and Mental Health, The University of Melbourne, Melbourne, VIC, Australia; 3 Department of Medicine, The University of Melbourne, Melbourne, VIC, Australia

**Keywords:** microelectrode array, all-optical interrogation, patterned photostimulation, multimodal neural interfaces, synaptic plasticity, network dynamics, neural computation

## Abstract

Understanding how synaptic interactions lead to circuit dynamics for neural computation requires experimental tools that can both observe and perturb neuronal activity across spatial and temporal scales. Microelectrode arrays (MEAs) provide scalable access to population spiking activity, yet they lack the spatial resolution and molecular specificity to precisely dissect synaptic mechanisms. In contrast, recent advances in optogenetic actuators, genetically encoded calcium and voltage indicators, and patterned photostimulation have transformed *in vitro* research, enabling all-optical interrogation of synaptic plasticity, functional connectivity, and emergent network dynamics. Further progress in transparent MEAs and hybrid optical–electrical systems has bridged the divide between electrophysiology and optical control, allowing simultaneous, bidirectional interaction with biological neural networks (BNNs) and real-time feedback modulation of activity patterns. Together, these multimodal *in vitro* platforms provide unprecedented experimental access to how local interactions shape global network behavior. Beyond technical integration, they establish a foundation for studying biological computation, linking mechanistic understanding of synaptic processes with their computational outcomes. This mini-review summarizes the progression from conventional MEA-based electrophysiology, through all-optical interrogation, to integrated multimodal frameworks that unite the strengths of both modalities.

## 1 Introduction

A major challenge in neuroscience lies in elucidating how collective neural circuit activity drives information processing and adaptive behavior (Bassett and Sporns, 2017). Bridging molecular synaptic mechanisms and emergent network-level functions requires experimental paradigms capable of both observing and perturbing circuit dynamics with precise spatial and temporal resolution (Frank et al., 2019; Liu et al., 2023). *In vitro* neuronal preparations, from dissociated cultures to brain slices and organoids, have long provided controlled environments for dissecting cellular and network mechanisms (Lv et al., 2023; Osaki et al., 2024). Recent technological progress has moved these preparations from passive observation toward active, closed-loop interrogation of plasticity, dynamics, and computation (Li L. et al., 2024), including pattern recognition (Shao et al., 2025), adaptive response (Kagan et al., 2022), and reservoir computing (Cai et al., 2023) demonstrated in recent work.

The introduction of microelectrode arrays (MEAs) marked a pivotal advance, enabling long-term, parallel monitoring of neuronal ensembles (Thomas et al., 1972; Obien et al., 2015). As discussed in Section 2, MEAs established the foundation for investigating network-level activity patterns and plasticity (Eytan and Marom, 2006). Their utility, however, is limited by a lack of cell-type specificity, insensitivity to subthreshold dynamics, and diffuse current spread during stimulation, which obscure mechanistic insight (Buzsáki, 2004). The development of complementary metal-oxide-semiconductor (CMOS) MEAs substantially improved spatial resolution, reaching densities above 20,000 electrodes per array (Schröter et al., 2025), yet issues such as electrode crosstalk and bandwidth constraints persist, as shown in our earlier studies (Habibollahi et al., 2023). Consequently, while MEAs excel at capturing population-level spiking, they cannot precisely resolve the underlying cellular interactions (Buzsáki, 2004).

Optical methods, particularly calcium imaging, offer superior spatial resolution and improved signal-to-noise performance for mapping network activity, as demonstrated in our recent studies (Sun et al., 2021; Sun et al., 2023; Sun et al., 2024a; Sun et al., 2024b). A paradigm shift in *in vitro* research occurred with the advent of all-optical interrogation, described in Section 3. By coupling optogenetic actuators with genetically encoded indicators, researchers can simultaneously manipulate and monitor defined neuronal populations with subcellular precision (<10 μm) (Deisseroth, 2011; Emiliani et al., 2015; Fernandez-Ruiz et al., 2022). This capability has transformed the field from correlational to causal investigation, revealing direct links between microcircuit connectivity, plasticity rules, and emergent computation (Jazayeri and Afraz, 2017; Sumi et al., 2023).

Electrophysiology and optical approaches have complementary strengths: the former captures ground-truth voltage signals at millisecond timescales, whereas the latter offers unparalleled access to genetically defined and spatially resolved populations (Ramezani et al., 2021). This complementarity has motivated the development of integrated multimodal interfaces that merge MEAs with optical stimulation and imaging, enabling concurrent electrical and optical interrogation (Buzsáki et al., 2015; Miccoli et al., 2019; Middya et al., 2021; Xu et al., 2024). Such hybrid systems permit real-time, closed-loop modulation of network activity, thereby allowing direct tests of how circuit dynamics encode and transform information. Although many multimodal technologies were originally developed *in vivo*, related concepts are now emerging *in vitro* (Shew et al., 2010; Yakushenko et al., 2013; Kshirsagar et al., 2019; Shaik et al., 2020; Middya et al., 2021; Shin et al., 2021), highlighting the growing feasibility of electrical–optical integration in culture.

**Figure 1:**
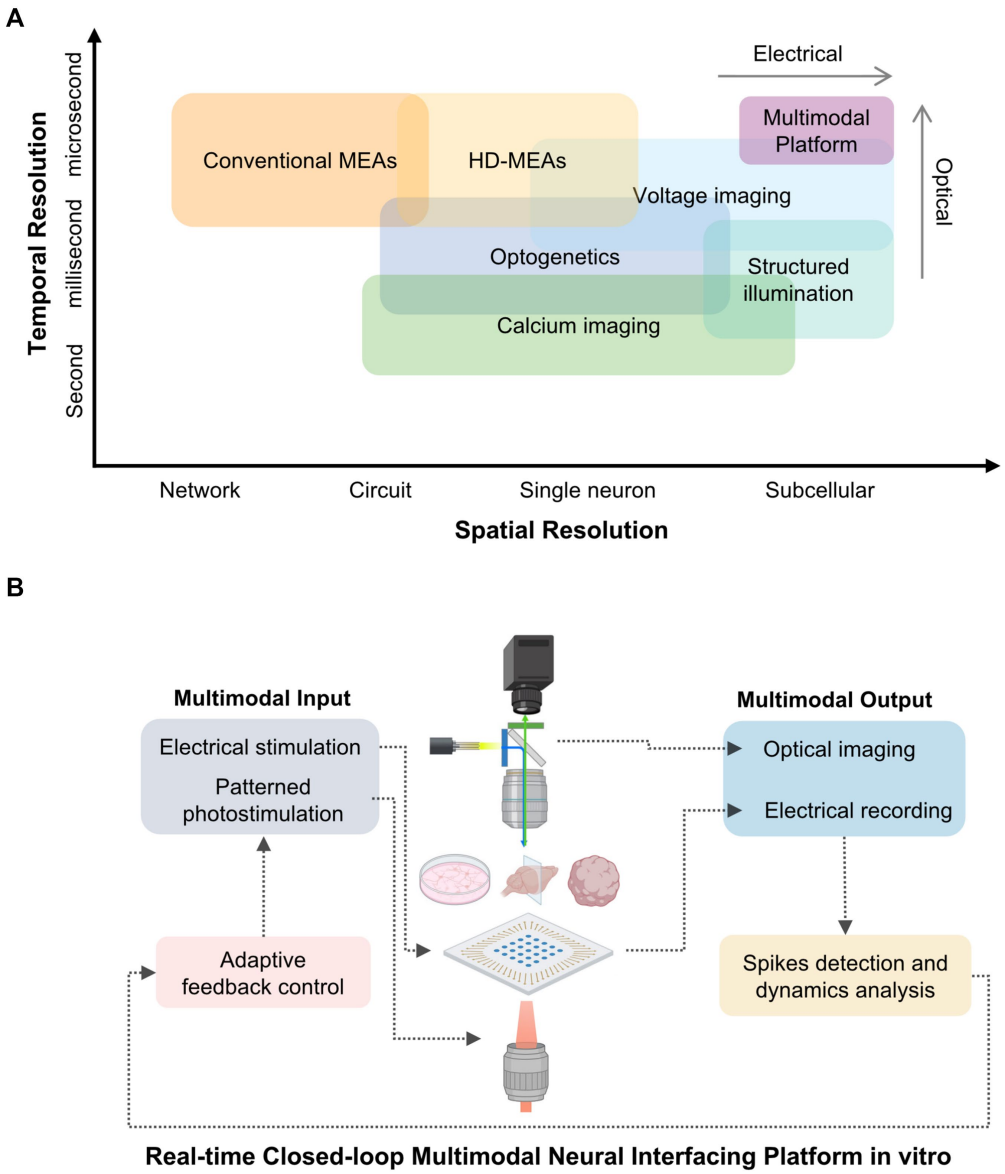
Comparative resolution and conceptual framework of multimodal in vitro neural interfacing platforms. **(A)** Spatial and temporal resolution of representative neural interfacing techniques. Electrical methods such as conventional and high-density microelectrode arrays (MEAs) provide millisecond-scale temporal fidelity but limited spatial resolution. Optical approaches, including calcium imaging and optogenetic stimulation, achieve single-cell or subcellular specificity at slower temporal rates. Voltage imaging bridges this gap with millisecond-resolved optical access, while structured-illumination strategies further enhance spatial precision. These techniques form the basis for multimodal platforms that integrate electrical and optical interrogation of biological neural networks (BNNs). **(B)** Conceptual schematic of an integrated multimodal platform combining electrical and optical modalities for bidirectional interrogation of BNNs. Electrical stimulation and patterned photostimulation deliver controlled inputs, whereas electrical recording and optical imaging yield complementary readouts. Real-time spike detection, dynamics analysis, and adaptive feedback close the loop, enabling precise modulation of network activity and investigation of activity-dependent plasticity.

This mini-review charts the conceptual and technological evolution of these approaches—from MEAs to all-optical systems and finally to integrated multimodal platforms (Figure 1). These innovations enable precise interrogation of vitro biological neural networks (BNNs), providing scalable and mechanistic models for probing how synaptic plasticity gives rise to computation and complex function.

## 2 Electrophysiological foundations: strengths, limitations, and legacy

The development of MEAs in the 1970s represented a foundational advance in in vitro neurophysiology, enabling simultaneous extracellular recordings from multiple neurons and laying the groundwork for scalable circuit-level investigations (Thomas et al., 1972). By embedding microelectrodes in a planar substrate, MEAs allowed minimally invasive, parallel recordings of extracellular spikes and local field potentials in cultured neurons and acute slices (Gross et al., 1977; Pine, 1980). This design enabled stable, long-term tracking of population activity, opening access to coordinated network dynamics previously beyond reach (Potter and DeMarse, 2001).

Initial MEA studies provided some of the first experimental demonstrations of network-level plasticity. Patterned electrical stimulation through MEA electrodes induced long-term potentiation (LTP) and depression (LTD), demonstrating that synaptic learning rules operate not only at individual connections but across neuronal ensembles (Jimbo et al., 1999). Later, spike-timing-dependent plasticity (STDP) paradigms demonstrated that precise temporal relationships between pre- and postsynaptic activity modulate circuit connectivity during development (Wagenaar et al., 2006). These findings extended classical synaptic principles into the mesoscale, linking cellular plasticity with emergent network behavior (Eytan and Marom, 2006).

However, these pioneering applications also revealed the limitations of electrode-based approaches, as shown in Figure 1A. MEAs detect only extracellular spikes but cannot access subthreshold or dendritic potentials essential for synaptic computation (Buzsáki, 2004). Although high-density CMOS MEAs approach micrometer-level spatial resolution, their non-planar surfaces are less compatible with microfabricated structures, such as PDMS microchannels, that require precise topography for axon guidance or modular network design (Duru et al., 2022). Moreover, extracellular signals lack inherent cell-type specificity: spikes from excitatory, inhibitory, or genetically defined neurons cannot be readily distinguished, limiting the interpretability of population activity and its mechanistic origins (Buzsáki et al., 2015). Electrical stimulation also suffers from current spread from the electrode tip, which activates a broad neuronal population beyond the target region (Yizhar et al., 2011). As a result, MEAs excel at tracking the timing and structure of network activity, but offer limited resolution into the cellular identities and synaptic mechanisms shaping those dynamics. These constraints also restrict the translational applications. In pharmacological studies, MEAs measure overall changes in the network, but cannot precisely resolve the receptor systems or intracellular pathways through which compounds act from electrophysiological data alone (McConnell et al., 2012; Saavedra et al., 2021). Similarly, developmental studies describe stereotyped patterns of network maturation, yet the absence of spatial and molecular specificity hinders mechanistic interpretation, such as identifying the contributions of receptor expression, synaptogenesis, or axon guidance (Lv et al., 2023).

In summary, MEAs established a durable and scalable platform for extracellular electrophysiology, and they remain a mainstay for long-term population-level recordings. However, their inherent spatial and functional limitations have motivated the pursuit of complementary techniques. Optical approaches have emerged to fill this gap, extending circuit interrogation beyond spiking activity to include underlying cellular and synaptic processes, and enabling the transition toward causal and multimodal paradigms (Emiliani et al., 2015).

## 3 All-optical interrogation: from synapses to networks

All-optical platforms have transformed *in vitro* circuit neuroscience by integrating genetic specificity, precise spatiotemporal control, and high-content imaging within a single experimental framework. Optogenetic actuators, such as channelrhodopsins and their red-shifted or fast-kinetic variants, enable temporally precise control of defined neuronal populations at the millisecond scale, minimal spectral crosstalk and reduced phototoxic effects (Kishi et al., 2022; Tanaka et al., 2024). Complementary optical reporters extend observation across scales: genetically encoded calcium indicators (GECIs) like the GCaMP family, report population-level calcium transients that correlate with spiking (Zhang Y. et al., 2023), whereas genetically encoded voltage indicators (GEVIs) provide access to fast subthreshold (millisecond to sub-millisecond) and dendritic voltage fluctuations (Bando et al., 2019) (Figure 1A).

Spatial precision is achieved through structured-illumination strategies (Figure 1). One-photon systems employing digital micromirror devices (DMDs), which use high-speed arrays of tiltable micromirrors to project programmable light patterns, support rapid patterned excitation across wide fields (Dudley et al., 2003; Zhu et al., 2012). In contrast, two-photon holographic photostimulation via spatial light modulators (SLMs) enables volumetric targeting of user-defined ensembles in three dimensions (Papagiakoumou et al., 2018). These stimulation approaches are increasingly coupled with advanced imaging techniques, like resonant-scanning multiphoton microscopy, a high-speed method using resonant galvanometer mirrors to achieve micrometer-level resolution recording across planes and populations (Prevedel et al., 2016; Hsu et al., 2023). Moreover, MAPSI (Miniscope with All-optical Patterned Stimulation and Imaging) (Zhang J. et al., 2023) recently demonstrates compact integration of calcium imaging and patterned photostimulation, building on advances from the open-source Miniscope project (Cai et al., 2016). Although developed for *in vivo* use, these platforms exemplify all-optical interrogation strategies that are inspiring modular and miniaturized approaches for *in vitro* applications.

Collectively, these developments establish a versatile experimental framework for probing synaptic plasticity, functional connectivity, and emergent circuit dynamics (Bassett and Sporns, 2017; Zhang et al., 2022). Although many of these applications have been extensively explored in vivo, in vitro platforms remain highly valuable, offering experimental control and scalability that continue to drive methodological innovation and mechanistic discovery.

### 3.1 Synaptic plasticity enabled by optical precision

All-optical approaches have enabled researchers to induce and monitor synaptic plasticity with subcellular accuracy, allowing direct examination of the mechanisms linking local activity to long-term changes in connectivity. By pairing temporally patterned photostimulation of pre- and postsynaptic partners with real-time imaging of calcium influx or spine morphology, it becomes possible to visualize how spatially constrained interactions translate into persistent synaptic modifications (Fan et al., 2023). Studies employing spine-targeted activation and high-resolution reporters have shown that the spatial organization of active spines, particularly their dendritic compartmentalization and clustering, shapes the outcome of plasticity (Cichon and Gan, 2015; Hayashi-Takagi et al., 2015). These results support the view that both the relative timing and the spatial arrangement of inputs jointly determine the likelihood and magnitude of potentiation, integrating Hebbian temporal rules with localized dendritic processing (Magee and Grienberger, 2020).

Choosing an appropriate optical reporter is critical for interpreting activity-dependent changes in neural circuits. Calcium imaging offers high-throughput readouts across large populations with favorable signal-to-noise ratios, but reflects voltage changes only indirectly and with limited temporal resolution (Ali and Kwan, 2019). By contrast, GEVIs provide a direct, temporally resolved measure of membrane potential, capturing subthreshold events that calcium signals typically miss (Knöpfel and Song, 2019; Villette et al., 2019). Because such subthreshold fluctuations often gate synaptic integration and influence plasticity induction, simultaneously capturing voltage and calcium dynamics allows a more complete mechanistic characterization. For instance, dual imaging has revealed compartment-specific summation rules, such as distinct dendritic integration profiles in cerebellar interneurons (Tran-Van-Minh et al., 2016). Although calcium transients correlate with various forms of plasticity, their sufficiency for causal inference remains underinvestigated. Increasingly, studies underscore the importance of ground-truth validation via simultaneous electrophysiology, particularly when interpreting subtle or distributed plasticity effects (Forli et al., 2021; Fan et al., 2023). This recognition has motivated the multimodal strategies outlined later in this review.

### 3.2 Functional connectivity mapping with patterned photostimulation

At the circuit level, structured optical stimulation provides a means to causally probe functional connectivity. By selectively activating or silencing genetically defined neuronal subsets, researchers can examine whether specific microcircuit motifs are necessary or sufficient to drive ensemble-level dynamics (Papaioannou and Medini, 2022; Hira and Isomura, 2025). Two-photon holography and other multi-site activation techniques allow the simultaneous control of spatially distributed neurons, while imaging population-wide calcium or voltage signals (Russell et al., 2022; Kim et al., 2023). These approaches have shown that brief stimulation of compact, topologically organized ensembles can evoke reproducible activity patterns, thereby linking synaptic connectivity with emergent population behavior.

Functional maps derived from stimulation–response relationships represent effective rather than anatomical connectivity, which can diverge substantially from synaptic wiring diagrams (Papaioannou and Medini, 2022). Artifacts from light scattering, opsin cross-activation, and cumulative phototoxicity limit the number of neurons that can be targeted simultaneously (Backhaus et al., 2023). To increase reproducibility and interpretability, ongoing efforts aim to standardize metrics such as spatial precision, false-positive detection rates, and cumulative exposure thresholds in chronic settings (Altahini et al., 2024).

As discussed further in the next section, integrating optical methods with high-density electrophysiology offers an orthogonal validation strategy. This enables testing whether optically evoked activity elicits the expected spike output, thereby refining inferences about functional connectivity and enhancing the reliability of network-level circuit mapping.

### 3.3 Network-scale dynamics and emergent computation *in vitro*

At the network scale, all-optical technologies support simultaneous observation and targeted control of distributed circuit activity, bridging the gap between synaptic plasticity and emergent ensemble dynamics. Wide-field calcium and voltage imaging techniques reveal how localized interactions among neurons give rise to global network states, including oscillations, wave propagation, and synchronization (Ren and Komiyama, 2021; Sun et al., 2023). These phenomena represent the integrated outcome of microcircuit activity and provide a quantitative framework for linking single-neuron plasticity with higher-order behavior. By introducing spatially patterned photostimulation, researchers can deliver precise input motifs, enabling causal tests of how structured stimuli are processed into population-level responses in both cultures and organoids (Sumi et al., 2023; Jieqiong et al., 2025).

Recent advances have pushed this approach further by enabling closed-loop optical control, in which real-time feedback dynamically modulates stimulation in response to ongoing activity patterns (Firfilionis et al., 2021). These experiments have been employed to stabilize desired network states, induce plasticity in targeted subpopulations, and explore the mechanisms by which subnetworks transition between attractor-like regimes (Newman et al., 2015). Combining online analysis with closed-loop manipulation facilitates causal dissection of information flow, while theoretical tools from graph and information theory help characterize effective connectivity and signal propagation *in vitro*.

Although simplified relative to *in vivo* systems, optically accessible in vitro models can reproduce core computational features, including recurrent loops, sparse coding, and activity-dependent network reorganization (Olshausen and Field, 1996; Abbott and Regehr, 2004; Douglas and Martin, 2004; Welkenhuysen et al., 2016; Bayat et al., 2022). By unifying observation, manipulation, and control within the same framework, all-optical strategies establish a mechanistic continuum from synaptic changes to global dynamics (Emiliani et al., 2015). This conceptual progression, from the local plasticity to emergent computation, lays the groundwork for multimodal integration, in which electrical and optical methods combine to support deeper, quantitative exploration of network function (Figure 1B).

## 4 Advancing neural interfacing with multimodal platforms

As shown in Figure 1B, integrating optical and electrophysiological modalities creates a cohesive platform for probing in vitro circuits across complementary domains of measurement and control (Kim et al., 2017; Ramezani et al., 2025) (Figure 1). While optical methods afford genetic specificity and volumetric access at submicron to micron spatial resolution, electrophysiology provides direct readout of voltage dynamics with sub-millisecond temporal precision. By co-registering both modalities within a unified spatial reference frame, perturbations and recordings can be aligned across techniques, enabling systematic comparisons between optically and electrically derived signals.

The following subsections trace the progression from parallel acquisition and cross-modal validation, through real-time feedback architectures for adaptive control, to closed-loop frameworks that interrogate learning rules and computational motifs (Alon, 2007). This progression illustrates how multimodal systems evolve from passive observation toward active modulation, ultimately supporting hypothesis-driven dissection of high-order function.

### 4.1 From concurrent recording to cross-modal validation

The integration of optical and electrical modalities has been propelled by advances in transparent MEAs, which resolve the longstanding trade-off between electrical interfacing and optical accessibility (Thunemann et al., 2018). Materials such as graphene, indium–tin oxide (ITO), and conductive polymers enable low-impedance yet optically transparent electrodes, supporting simultaneous high-resolution fluorescence imaging, using either two-photon or wide-field method, and electrical recording from the same neuronal population (Liu et al., 2018; Li et al., 2023; Shankar et al., 2025).

Concurrent acquisition of optical and electrical datasets is critical for cross-modal validation, ensuring that genetically encoded indicators faithfully reflect the underlying electrophysiological activity (Grienberger et al., 2022; Xu et al., 2024). Comparative analyses between calcium transients and spike trains captured by high-density MEAs have defined the temporal and amplitude limitations of optical indicators: calcium signals act as intrinsic low-pass filters, smoothing high-frequency bursts and obscuring precise spike timing (Yger et al., 2018; Wei et al., 2020). Establishing electrical ground truth is thus essential for calibrating and quantitatively interpreting optical data (Panzeri et al., 2017).

**Figure 2:**
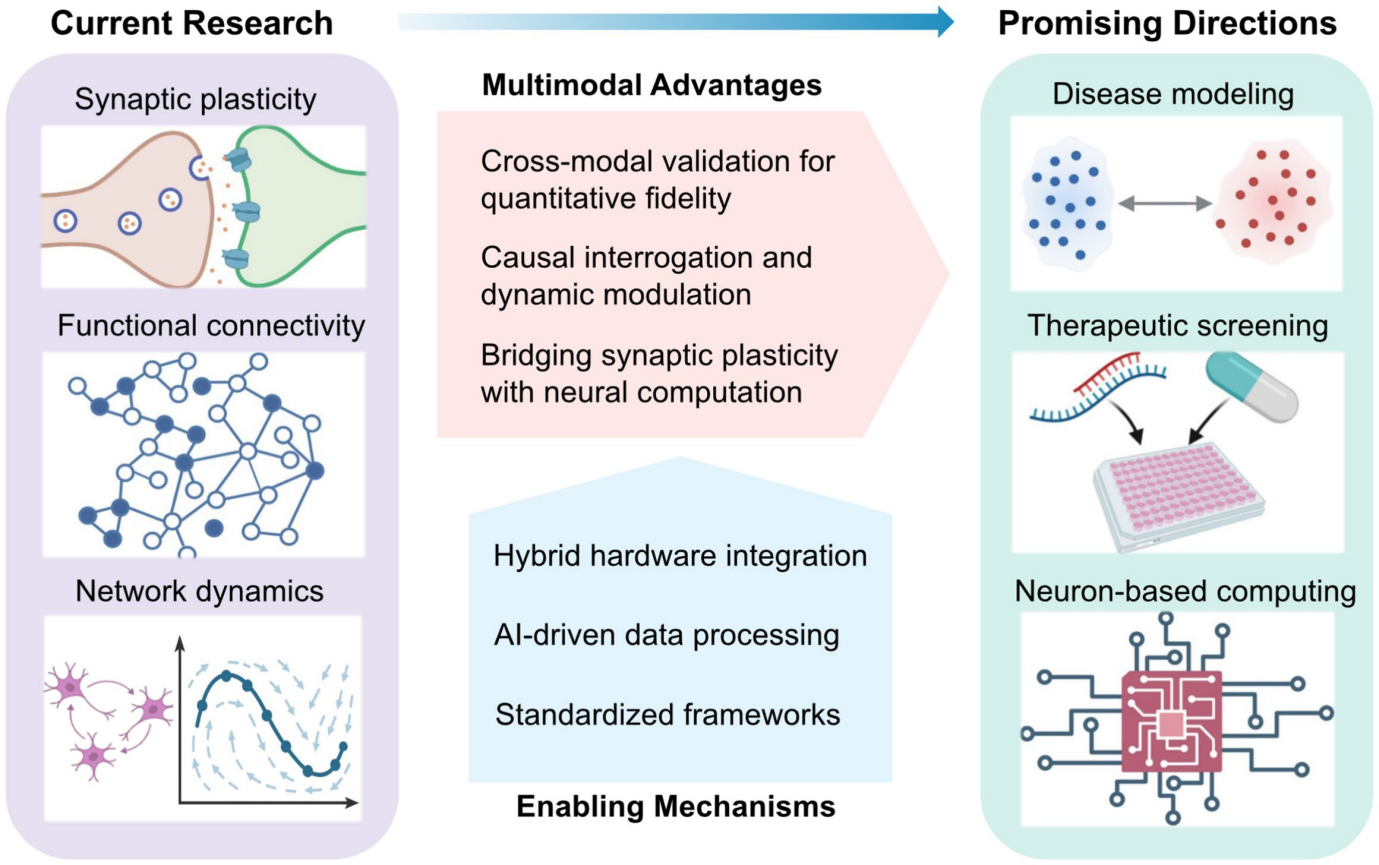
Conceptual landscape of multimodal in vitro neural interfacing: current research, enabling mechanisms, and emerging directions. Current research focuses on the integration of electrical and optical modalities to investigate synaptic plasticity, functional connectivity, and emergent network dynamics. The multimodal approach confers key advantages—including quantitative cross-validation, causal and adaptive control, and the bridging of synaptic mechanisms with network-level computation. These advances are underpinned by key enabling mechanisms such as optical-electrical hardware integration, AI-driven data processing, and standardized experimental frameworks. Collectively, these platforms are converging toward disease modelling, neurotherapeutic development, and neuron-based computing, positioning multimodal systems as a foundation for next-generation translational neurotechnology.

Such cross-validation elevates multimodal platforms beyond descriptive imaging, enabling mechanistic inference (Figure 2). For instance, optogenetic activation of defined excitatory neurons can trigger population-wide responses, whose propagation is resolved with millisecond precision across thousands of electrodes, linking local microcircuit activity to emergent network behavior (Müller et al., 2015; Kobayashi et al., 2024). Conversely, electrical stimulation can target inhibitory circuits previously identified via optical mapping (Dadarlat et al., 2024). Yet these bidirectional paradigms pose analytical challenges, particularly in co-registering optical and electrical coordinate systems, and in constructing models that jointly capture fluorescence dynamics and extracellular voltage signals to infer latent states of the network. Recent developments in miniaturized integrated devices have begun to address these limitations, opening new avenues for precise and comprehensive investigation of circuit dynamics (Wu et al., 2015; Tian et al., 2022).

### 4.2 Real-time closed-loop platforms for dynamic control

The inherent ability of multimodal systems to both record and manipulate neural activity at the cellular level makes them ideally suited for closed-loop architectures. In electrical–optical platforms, extracellular spikes are captured by the electrode array while calcium or voltage imaging provides single-cell–resolved optical readout; in parallel, patterned optogenetic stimulation enables targeted activation of individually identified neurons (Shew et al., 2010; Zhang et al., 2018; Ramezani et al., 2024). In such setups, sensing and stimulation components are integrated into a unified feedback loop. By co-localizing readout and actuation, these platforms can decode neural activity in real time and adjust stimulation parameters to guide the network toward defined target states. Leveraging graphics processing unit (GPU)-accelerated imaging pipelines and field programmable gate array (FPGA)-based controllers, state-of-the-art closed-loop systems achieve latencies as low as tens of milliseconds, which is sufficient to engage intrinsic oscillations and spike-timing-dependent plasticity (Park et al., 2017; Chen et al., 2023).

Such systems have been deployed to modulate circuit dynamics across diverse *in vitro* preparations, from dissociated cultures to brain organoids. Both optical and electrical feedback loops have been shown to stabilize firing rates, suppress epileptiform-like discharges (Wagenaar et al., 2005; Newman et al., 2015), and promote the self-organization of functional connectivity in human organoids (Osaki et al., 2024). Emerging efforts aim to transition from heuristic control rules to model-based predictive frameworks, which proactively shape activity patterns to induce desired plasticity or computational motifs (Saponati and Vinck, 2023; Mehta et al., 2024).

The complexity of hybrid optical–electrical systems demands standardized and transparent experimental reporting to ensure reproducibility and facilitate cross-laboratory comparisons (Ramezani et al., 2021) (Figure 2). Key performance metrics, including stimulation latency distributions, optical–electrode registration accuracy, and total optical dose or injected charge, should be consistently reported. Simultaneous electrical recordings remain essential for calibrating optical signals and differentiating genuine biological variability from system-level artifacts (Qiang et al., 2018). As these multimodal frameworks evolve, they are laying the foundation for investigating how adaptive control principles are instantiated in biological intelligence systems.

Capitalizing on these capabilities for real-time modulation, the next step is to test whether performance-contingent feedback can drive self-organized improvement within BNNs—an inquiry that bridges engineering control with biological computation.

### 4.3 Probing biological computation in cultured networks

The same architectures used for dynamic control can be strategically repurposed to investigate learning and adaptive computation in living neural circuits. When stimulation is made contingent on network performance, feedback rules analogous to reinforcement learning paradigms can be implemented directly within the biological substrate, allowing activity-dependent modification of synaptic connectivity (Tessadori et al., 2012; Wülfing et al., 2019). Landmark demonstrations have shown that dissociated cortical cultures can acquire goal-directed behaviors, such as controlling a simplified Pong task, when provided with structured sensory feedback linking performance outcomes to patterned electrical stimulation (Kagan et al., 2022). Subsequent work has further demonstrated that iterative training paradigms can enhance the pattern recognition and discrimination capabilities of cultured BNNs (Shao et al., 2025).

These experiments establish dissociated neuronal assemblies as embodied adaptive systems, offering direct tests of how neural plasticity underlies learning and information processing. Such platforms have been exploited to examine how intrinsic neuronal heterogeneity contributes to generalization within reservoir computing frameworks (Jaeger and Haas, 2004; Tanaka et al., 2019; Yada et al., 2021; Sumi et al., 2023) and to investigate how spontaneous activity reorganizes into predictive patterns through unsupervised reconfiguration (Yaron et al., 2025). Extending these paradigms from two-dimensional cultures to three-dimensional organoids represents a key frontier for modeling higher-order circuit adaptation and long-range connectivity (Cai et al., 2023).

Ultimately, the convergence of optical specificity, electrical fidelity, and real-time algorithmic control is transforming *in vitro* neural systems into standardized experimental platforms (Figure 2). Developing quantitative benchmark tasks and reproducible performance metrics will enable systematic cross-platform comparisons of learning capacity across biological preparations and feedback architectures. In this emerging framework, cultured networks are viewed not merely as simplified models of brain circuits, but as hybrid bio-computational systems in which neuronal plasticity and algorithmic learning interact to generate adaptive behavior—bridging the mechanistic studies of synaptic dynamics with the theoretical principles of computation.

## 5 Conclusion and outlook

The progression from planar MEAs to all-optical interrogation and, ultimately, to integrated multimodal systems represents a pivotal shift in neural interfacing. The field has evolved from the correlational, spike-based population analyses enabled by MEAs to precise, cell-type-specific interrogation achieved through optical techniques. By fusing the temporal fidelity of electrophysiology with the spatial and genetic targeting of optogenetics and imaging, current platforms define a new experimental paradigm. This convergence has redefined *in vitro* models: from passive observation platforms to interactive, closed-loop environments capable of simultaneously sensing and manipulating neural activity in real time (Ramezani et al., 2021).

Looking forward, as outlined in Figure 2, this multimodal convergence will depend on three core enabling mechanisms, spanning hardware integration, computational analysis, and standardized experimental frameworks for translational research. On the hardware side, a key challenge is seamlessly integrating high-density electrode arrays with optical access while maintaining stable, low-noise performance. Advances in transparent electrode materials have demonstrated strong potential for simultaneous imaging and electrophysiology, yet optimizing transparency, impedance, durability, and fabrication scalability remains an active area of research (Qiang et al., 2018; Thunemann et al., 2018; Yang et al., 2024; Shankar et al., 2025).

From a translational standpoint, multimodal platforms are well positioned to reshape disease modeling and therapeutic screening (Wang et al., 2022). Integrating multimodal tools with patient-derived iPSC neurons and brain organoids enables the creation of in vitro models that approximate circuit-level dysfunctions underlying neurological and psychiatric disorders (Lv et al., 2023; Birtele et al., 2025). Such preparations offer controlled yet scalable context for dissecting disease mechanisms and linking pharmacological perturbations to changes in network function, though issues of variability and reproducibility still limit large-scale applications (Saavedra et al., 2021).

Complementing the experimental advances, progress will also rely on improving analytical pipelines. High-dimensional multimodal datasets require both established methods, such as spike sorting, dimensionality reduction, and functional connectivity analysis (Cunningham and Yu, 2014; Bastos and Schoffelen, 2016; Pachitariu et al., 2024), and emerging machine-learning and AI-driven approaches for state estimation and cross-modal data fusion (Saxe et al., 2021; Hong et al., 2024). The development of these computation tools will enable robust interpretation of neural activity across scales and modalities, maximizing the scientific yield of multimodal platforms.

Still, few studies have achieved the full integration of (i) bidirectional electrical–optical interfacing, (ii) simultaneous patterned stimulation and multimodal recording, (iii) in vitro system implementation, and (iv) closed-loop feedback (Xu et al., 2024; Ramezani et al., 2025). Realizing all four elements would complete the loop between perturbation and readout, offering a coherent framework for testing hypotheses about neural computation. Such a framework opens new territory at the interface of neuroscience and engineering, enabling direct investigation of how neural circuits adapt to structured feedback, reorganize connectivity, and develop predictive, goal-directed dynamics. Extending these paradigms to more complex organoid architectures may reveal how BNNs realize reinforcement and generalization—a cornerstone of adaptive learning capability (Neftci and Averbeck, 2019; Wülfing et al., 2019; Li Q. et al., 2024).

Taken together, multimodal in vitro systems are no longer solely experimental tools, but rather serve as both investigative platforms for fundamental neuroscience and living substrates for neuron-based computation. As such, they represent a critical inflection point: one in which mechanistic understanding, computational modeling, and physical embodiment converge within a unified experimental framework.
